# Anatomy of Cowper’s gland in humans suggesting a secretion and emission mechanism facilitated by cooperation of striated and smooth muscles

**DOI:** 10.1038/s41598-021-96130-z

**Published:** 2021-08-18

**Authors:** Satoru Muro, Janyaruk Suriyut, Keiichi Akita

**Affiliations:** 1 Department of Clinical Anatomy, Tokyo Medical and Dental University (TMDU), 1-5-45 Yushima, Bunkyo-ku, Tokyo, 113-8510 Japan; 2 Department of Anatomy, Faculty of Medicine, Srinakharinwirot University, Bangkok, 10110 Thailand

**Keywords:** Gonads, Urethra, Anatomy, Urology

## Abstract

This study presents the detailed anatomy of the Cowper’s gland in humans. Elucidating the mechanism of secretion and emission of the Cowper’s gland requires analysis of the muscles around the Cowper’s gland. We hypothesized that the Cowper’s gland involves not only smooth muscle but also the striated muscles of the pelvic floor. Here, we provide comprehensive and three-dimensional anatomy of the Cowper’s gland and its surrounding structures, which overcomes the current local and planar understanding. In this study, seven male corpses of body donors were used to conduct macroscopic anatomy, histology, and three-dimensional reconstruction. The Cowper’s gland was surrounded laterally and posterosuperiorly by striated and smooth muscles, respectively. The striated muscle bundle was connected from the superficial transverse perineal muscle, levator ani, and external anal sphincter to the external urethral sphincter (rhabdosphincter). The smooth muscle was part of the deep transverse perineal muscle and entered between the bilateral Cowper’s glands and lobules. Our findings indicate that the secretion and emission of the Cowper’s gland in humans are carried out through the cooperation of striated and smooth muscles.

## Introduction

The Cowper’s gland (CG), which is also known as the bulbourethral gland, is a small, round structure located posterolateral to the membranous urethra in the deep perineal pouch. Secretions from the CG are responsible for lubricating the urethral lumen and neutralizing acidic urine^1^. Although it is generally believed that smooth muscle is involved in glandular secretion, a recent physiological study using rats reported that the striated muscle surrounding the CG was involved during secretion. This study showed that secretion from the CG involves not only the autonomic control of smooth muscle, but also the somatic control of striated muscle^2^. However, the mechanism of secretion and emission from the CG remains unclear in humans. The analysis of the muscles around the CG is important for elucidating the mechanism of secretion and emission from the CG.

There is a controversy regarding the anatomy of the muscles around the CG in human anatomy textbooks. Some report that the CG is surrounded by the external urethral sphincter (EUS) (urinary sphincter or rhabdosphincter), whereas others report that it is surrounded by the deep transverse perineal muscle (DTP)^3–6^. Furthermore, previous studies have pointed out that the EUS and DTP are difficult to analyze, and that textbook descriptions are inconsistent^7,8^. There is still a debate concerning the existence and composition of the EUS and DTP^9–11^. Thus, the detailed anatomy of the muscles surrounding the CG remains unclear.

Our research group has clarified that the external anal sphincter (EAS), superficial transverse perineal muscle (STP), and levator ani (LA) extend and surround the anterior and lateral sides of the urethra to form the EUS^12^. This means that the EUS is not an independent muscle, but a continuous structure with other pelvic floor muscles. In addition, we reported that the DTP was smooth muscle that was continuous with the longitudinal muscle of the rectum. This report provides a new interpretation of the DTP, which is classically considered a striated muscle.

We have reported the characteristic structure of the pelvic floor, in which the striated muscles share their muscle bundles and are continuous, and the smooth muscle occupies the space between them^11–20^. These studies suggest that striated and smooth muscles coordinate in anal function and pelvic floor support. This new anatomical understanding of the pelvic floor muscles could be applied to the study of the CG. The present anatomical study aimed to clarify the structure of the muscles around the CG in humans.

## Materials and methods

The seven male corpses of body donors used in this study were donated to our department. The donation document format was congruent with the Japanese law entitled, “Act on Body Donation for Medical and Dental Education.” All the donors had voluntarily declared, before their deaths, that their remains would be donated as materials for education and study. At that time, the purpose and method of using the corpses of body donors were explained, and consent was obtained. After their death, we explained the consent of the deceased to the bereaved families and confirmed in writing that there was no opposition. In addition, we are opting out by posting posters regarding the use of this research and the publication of research results. All corpses of body donors were fixed by arterial perfusion with 8% formalin and preserved in 30% alcohol. The corpses of body donors with a history of abnormalities in the pelvis were not used in the study. Details of body donors are shown in Table 1. Study approval was obtained from the Board of Ethics at Tokyo Medical and Dental University (approval number: M2018-006). All methods in the study were carried out in accordance with relevant guidelines and regulations.

**Table 1:** Details of body donors.

Age at death (years)	Sex	Cause of death	Medical history
60	Male	Kidney cyst infection	Polycystic kidney disease and chronic kidney disease
64	Male	Metastatic bone tumor	Appendicitis, varicose veins, and thalamic bleeding
74	Male	Brain embolism	Atrial fibrillation
75	Male	Lung cancer	Right middle and lower lobe resection, right 8th rib fracture, right forearm and left lower leg contusion, 1st lumbar compression fracture, sacral fracture, cervical spinal cord injury, left scapula fracture, and left 4th rib fracture
89	Male	Pneumonia	Influenza
91	Male	Senility	Kidney cancer
92	Male	Pneumonia	Chronic kidney disease

### Macroscopic anatomy

Three male corpses of body donors (ages at death: 64, 75, and 89 years) were used for macroscopic examination. The pelvis was obtained en bloc, and the muscles and connective tissue were sequentially dissected from the perineal side. The spatial relationship between the muscles and the CG was observed. Initially, the skin and subcutaneous tissue were removed, and the bony structures (the pubic and ischial bones) and perineal muscles were identified. Thereafter, the perineal muscles were gradually removed to reveal the perineal membrane, which was then detached to reveal the structures in the deep perineal pouch, including the muscles surrounding the CG.

### Histology

The pelvises of the other four male corpses of body donors (ages at death: 60, 74, 91, and 92 years) were used for histological examination. Two pelvises were frozen at − 80 °C and cut into 8-mm-thick transverse or coronal sections, from which small blocks for histological analysis were obtained. The blocks were fixed in 10% formalin, dehydrated, embedded in paraffin, and sectioned into 5-μm-thick sections. The region posterior to the bulb of the penis was isolated from the other two pelvises to generate histological specimens along the serial transverse or coronal plane for detailed observation. The tissue was embedded in paraffin and serially sectioned into 5-μm-thick specimens at 1-mm intervals.

The histological sections were stained with Masson’s trichrome or Elastica Masson to identify the muscular and connective tissues. Anti-smooth actin (ready-to-use actin, smooth muscle Ab-1, clone 1A4; Thermo Fisher Scientific, Fremont, CA) and anti-skeletal myosin (ready-to-use myosin, skeletal muscle Ab-2, clone MYSN02; Thermo Fisher Scientific) were used for immunological staining to confirm the distribution of smooth and striated muscle fibers. The detailed procedures have been described previously^11,13,15–17,20^.

### Three-dimensional reconstruction

The CG and surrounding muscles were analyzed using computer-assisted three-dimensional reconstruction. Three-dimensional reconstructions were made from the histological serial transverse sections, which were obtained from one of the samples subjected to histological examinations (age at death: 91 years), as described above. All 98 serial sections were scanned, and the structures (CG, striated muscles, smooth muscles, urethra, and prostate) were traced and colored. Section sequences were reconstructed using the SrfII software (ver. R.11.00.00.0-H, Ratoc, Tokyo, Japan, http://www.ratoc.com/eng/index.html). This technique is similar to that used in our previous reports^17,21^.

### Ethical approval

The study was approved by the ethics committee of Tokyo Medical and Dental University (approval number: M2018-006).

### Statement

All methods in the study were carried out in accordance with relevant guidelines and regulations.

## Results

### Macroscopic anatomy

**Figure 1:**
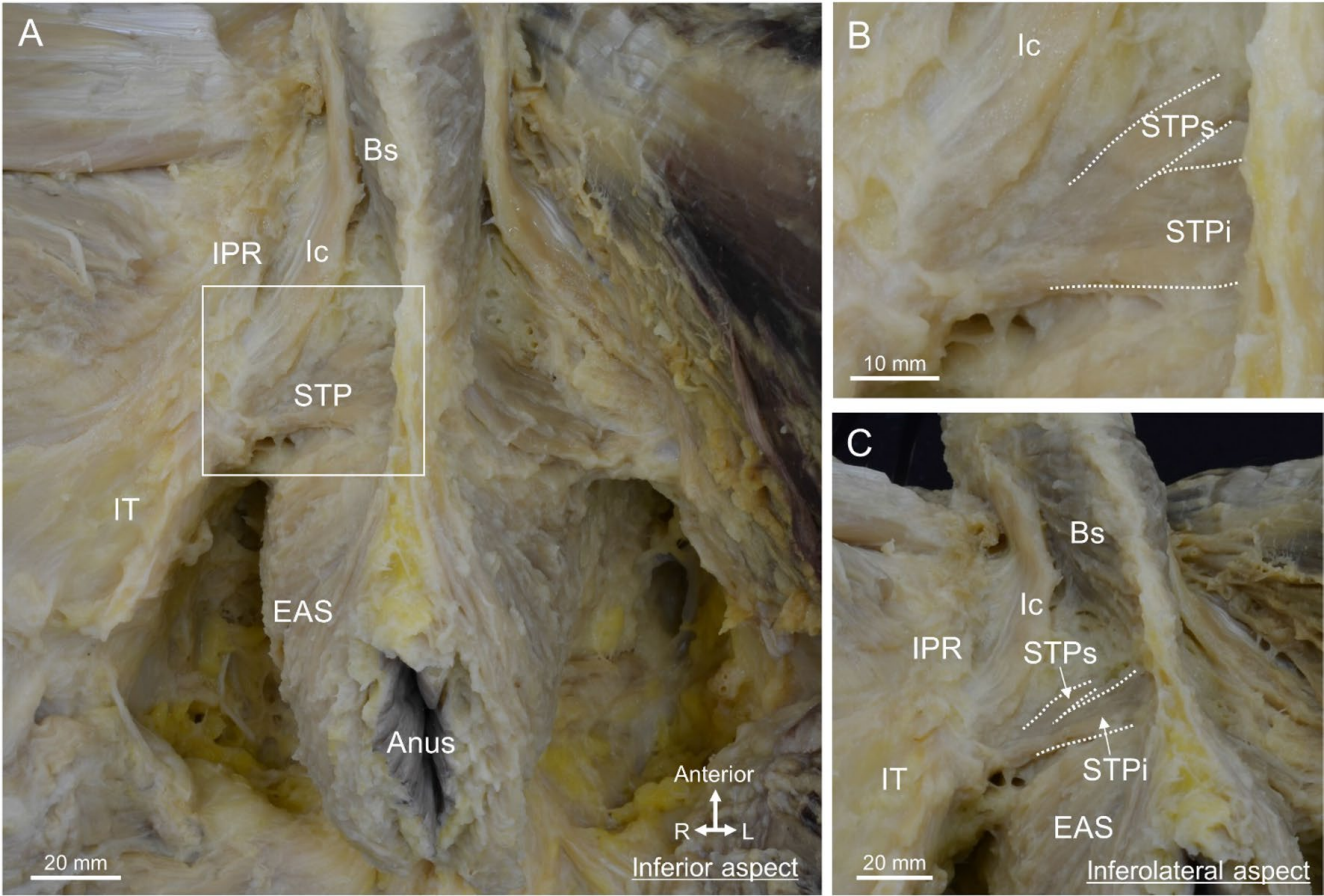
The central region of the male perineum where the perineal muscles gather. (**A**) The inferior aspect of the perineum, including the Bs, Ic, STP, and EAS muscles. (**B**) The magnified view of the rectangular space in (**A**). The STP was medially divided into two bundles, the STPs and STPi. (**C**) The inferolateral aspect, in which the pelvis was slightly tilted. The Bs and EAS were observed to be continuous. The Bs, EAS, and STP were concentrated in the central region of the perineum. *Bs* bulbospongiosus, *EAS* external anal sphincter, *Ic* ischiocavernosus, *IPR* ischiopubic ramus, *IT* ischial tuberosity, *STP* superficial transverse perineal muscle, *STPi* inferior bundle of STP, *STPs* superior bundle of STP.

The male pelvis was dissected from the perineal side, and the perineal muscles were observed (Fig. 1A). The bulbospongiosus (Bs) surrounded the bulb of the penis, the ischiocavernosus ran along the ischiopubic ramus, and the EAS surrounded the anal canal. The STP was laterally attached to the ischial tuberosity and medially divided into superior and inferior muscle bundles: the superior bundle of the STP (STPs) and inferior bundle of the STP (STPi) (Fig. 1B). When the pelvis was slightly tilted and viewed from the inferolateral aspect, the Bs ran anterosuperior to inferoposterior, and was continuous with the muscle bundle extending from the EAS (Fig. 1C). Because the muscle bundles of the STP approached medially, the central region of the perineum was the area where the Bs, STP, and EAS met.

**Figure 2:**
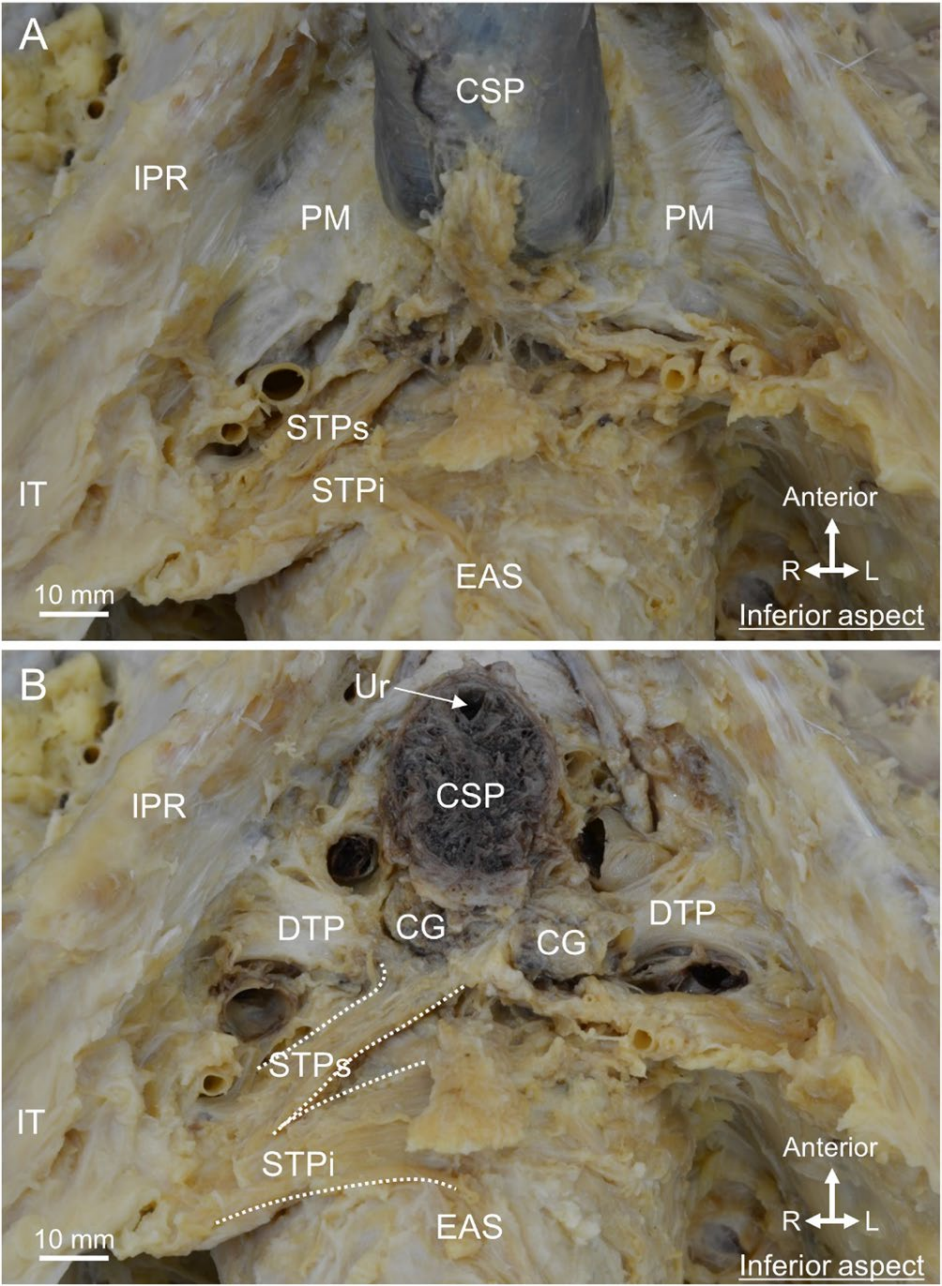
The Cowper’s gland and surrounding muscles. (**A**) After removal of the Bs and Ic from Fig. 1, the PM was observed on the left and right sides of the CSP and was attached to the bilateral IPR. The STPi ran superficially and connected to the EAS medially, whereas the STPs ran deeply and extended deeper than the PM. (**B**) After removal of the PM to reveal structures in the deep perineal pouch. The CG was observed posterior to the CSP. The STPs extended to around the CG. The DTP was deeper than the CG. *Bs* bulbospongiosus, *CG* Cowper’s gland, *CSP* corpus spongiosum penis, *DTP* deep transverse perineal muscle, *EAS* external anal sphincter, *Ic* ischiocavernosus, *IPR* ischiopubic ramus, *IT* ischial tuberosity, *PM* perineal membrane, *STP* superficial transverse perineal muscle, *STPi* inferior bundle of STP, *STPs* superior bundle of STP, *Ur* urethra.

The ischiocavernosus and Bs were removed to reveal the corpus spongiosum of the penis (CSP) and the perineal membrane (Fig. 2A). The perineal membrane consisted of firm connective tissue located on the left and right sides of the CSP, and attached to the bilateral ischiopubic rami. The STP ran transversely posterior to the perineal membrane (Fig. 2A). The STPi ran superficially and was connected to the EAS medially. STPs extended deeper than the perineal membrane superiorly. Thereafter, the perineal membrane was removed to reveal the structure of the deep perineal pouch (Fig. 2B). Bilateral CGs were observed posterior to the CSP. The STPs extended around the CG. In addition, a plate-like structure was observed deep to the CG. This plate-like structure had transverse fibers, and was laterally attached to the bilateral ischiopubic rami, suggesting that it was the DTP. Thus, the CG was surrounded by the STPs and DTP.

### Histology

**Figure 3:**
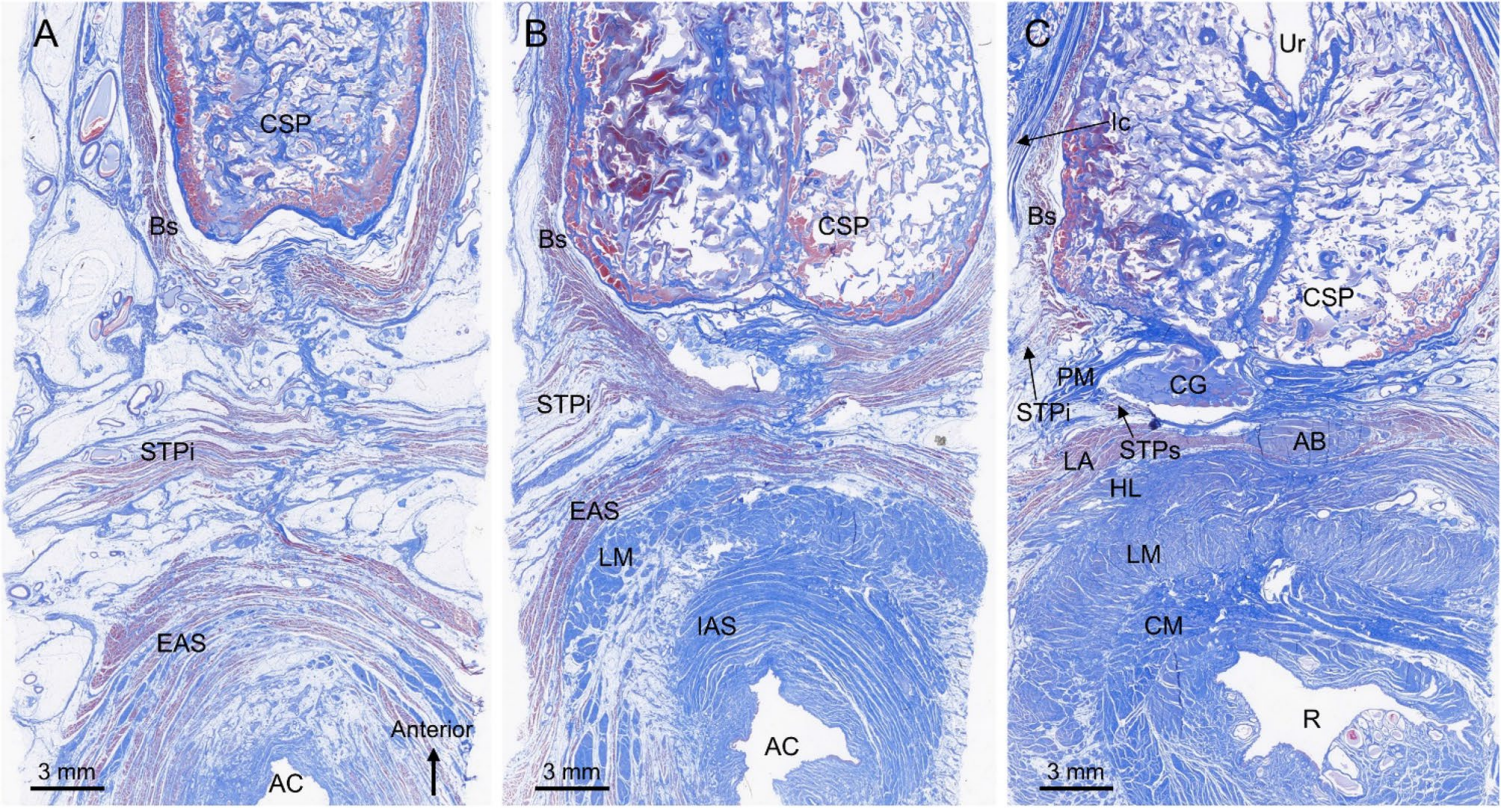
Transverse histological sections of the male perineum stained with Masson’s trichrome. (**A**) A superficial section in which the Bs, STPi, and EAS were observed. The STPi ran transversely between the Bs and EAS. (**B**) The Sect. 8 mm from the superior side of panel A in which the STPi was slightly displaced anteriorly and merged with the Bs. (**C**) The Sect. 8 mm from the superior side of panel B in which the STPi was displaced further anteriorly and integrated with the Bs. The CG was observed posterior to the CSP. The muscle fibers of the STPs were partially visible lateral to the CG. *AB* anterior bundle of LM, *AC* anal canal, *Bs* bulbospongiosus, *CG* Cowper’s gland, *CM* circular muscle, *CSP* corpus spongiosum penis, *EAS* external anal sphincter, *HL* hiatal ligament, *IAS* internal anal sphincter, *Ic* ischiocavernosus, *LA* levator ani, *LM* longitudinal muscle, *PM* perineal membrane, *R* rectum, *STP* superficial transverse perineal muscle, *STPi* inferior bundle of STP, *STPs* superior bundle of STP, *Ur* urethra.

Figure 3 shows transverse sections of the male perineal region. The Bs, STPi, and EAS were observed superficially (Fig. 3A). The Bs surrounded the lateral and posterior sides of the CSP, and the EAS surrounded the anal canal. The STPi ran transversely between the Bs and EAS. In the section 8 mm from the superior side of the section in Fig. 3A, the STPi was slightly displaced anteriorly and merged with the Bs (Fig. 3B). Around the anal canal, the longitudinal muscle and internal anal sphincter were observed internally to the EAS. In the section 8 mm from the superior side of the section in Fig. 3B, the STPi was displaced further anteriorly and integrated with the Bs. In this section, the muscle fibers of the Bs and STP did not extend to the midline, and only covered the lateral surface of the CSP (Fig. 3C). In the paramedian, the CG was posterior to the CSP, and muscle fibers of the STPs were observed lateral to CG.

**Figure 4:**
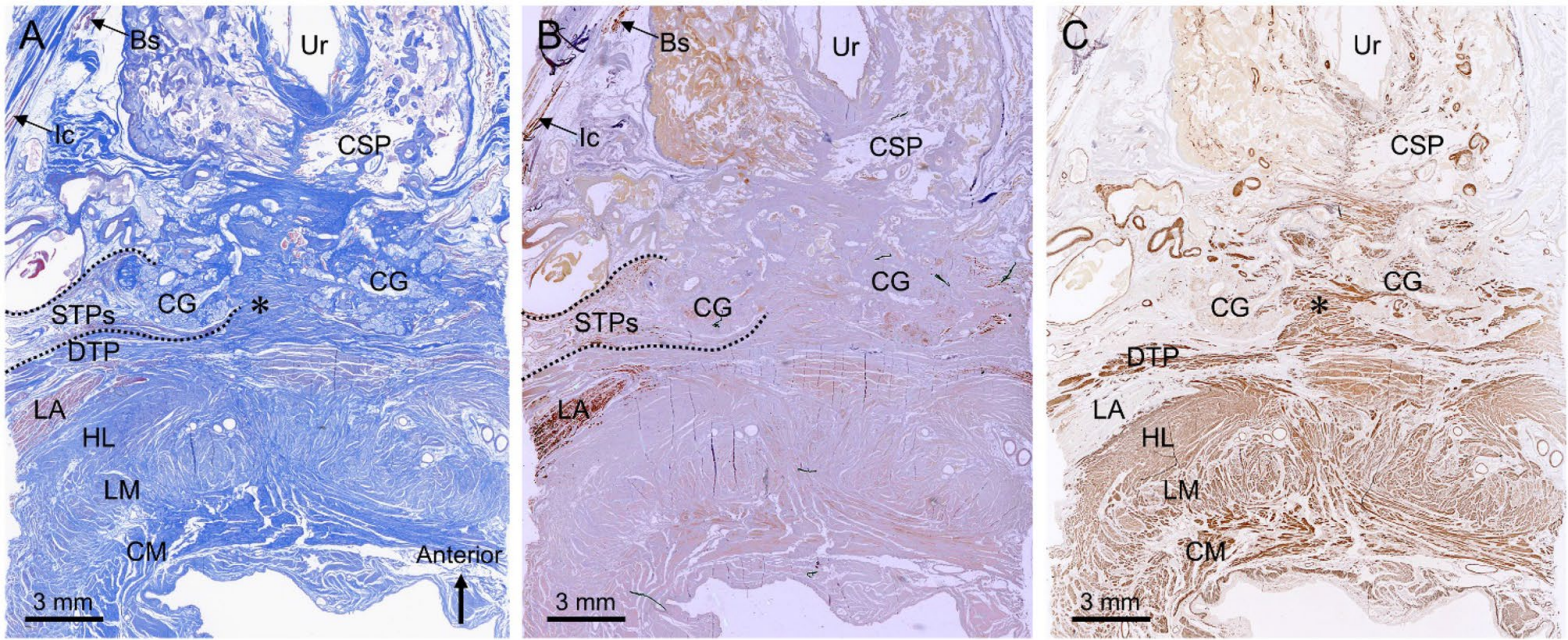
Transverse sections of the Cowper’s gland and surrounding muscles. (**A**) The Sect. 4 mm from the superior side of Fig. 3C, stained with Masson’s trichrome, in which the CG was observed posterior to the CSP. The STPs and DTP were located lateral and posterior to the CG. (**B**) Immunostaining for striated muscle. The striated muscle fibers of the STPs covered the lateral side of the CG. (**C**) Immunostaining for smooth muscle. The DTP consisted of smooth muscle and formed plate-like wall posterior to the CG. Its smooth muscle fibers extended between the bilateral CGs and between their lobules (asterisk). *Bs* bulbospongiosus, *CG* Cowper’s gland, *CM* circular muscle, *CSP* corpus spongiosum penis, *DTP* deep transverse perineal muscle, *HL* hiatal ligament, *Ic* ischiocavernosus, *LA* levator ani, *LM* longitudinal muscle, *STP* superficial transverse perineal muscle, *STPs* superior bundle of the STP, *Ur* urethra.

In the section 4 mm from the superior side of the section in Fig. 3C, the CG was observed posterior to the CSP (Fig. 4A). The STPs and DTP were located lateral and posterior to the CG. The striated muscle fibers of the STPs covered the lateral side of the CG (Fig. 4A,B). In addition, the thin layer of the striated muscle wrapped not only the lateral side but also the posterior side of the CG. The DTP consisted of smooth muscle and formed a plate-like wall posterior to the CG (Fig. 4A,C). Its smooth muscle fibers extended between the bilateral CGs, and between their lobules (indicated by the asterisk in Fig. 4C). Thus, the CG was surrounded laterally by striated muscle fibers of the STPs and posteromedially by the smooth muscle of the DTP.

**Figure 5:**
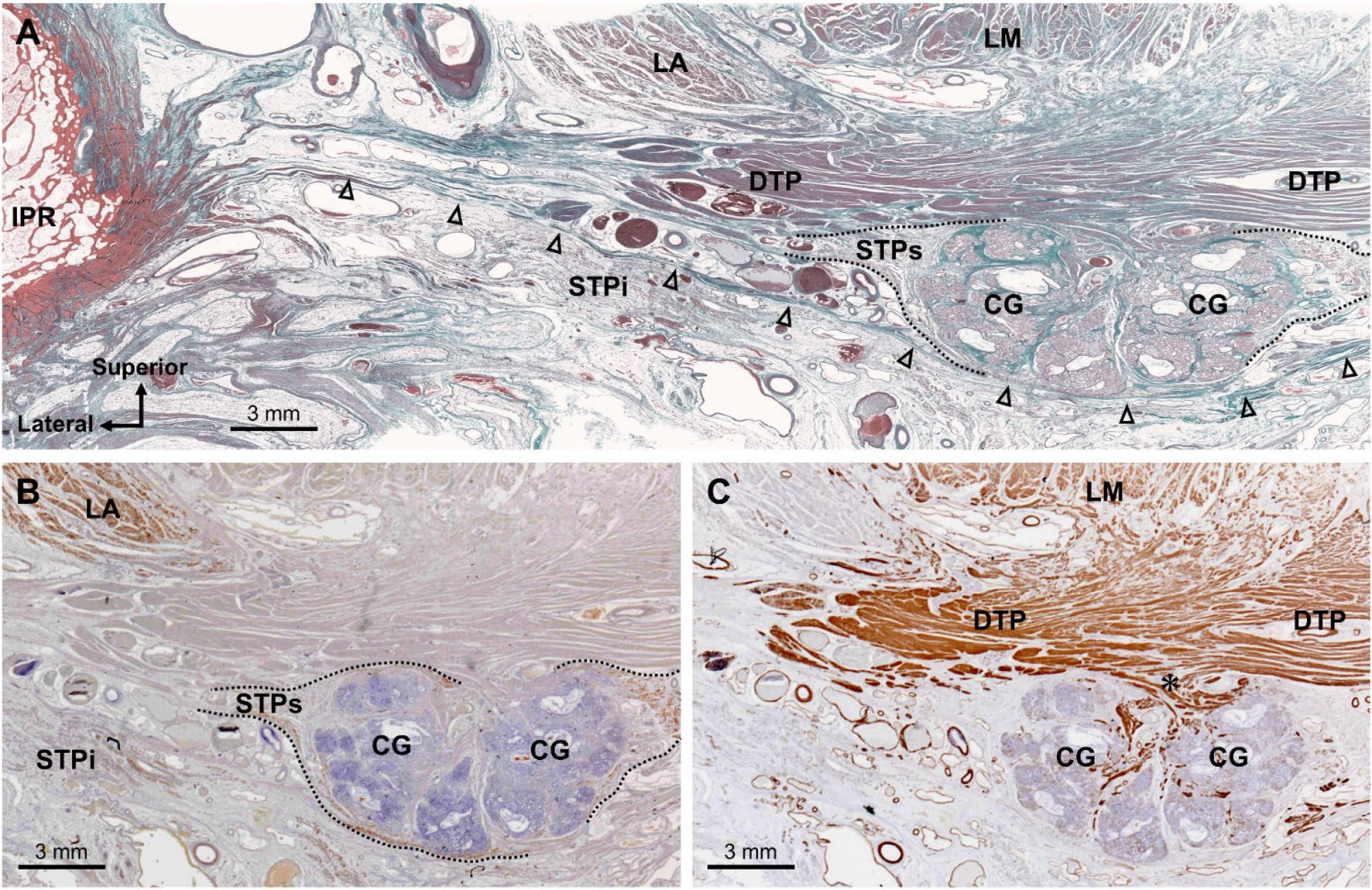
Coronal sections of the Cowper’s gland and surrounding muscles. (**A**) A coronal section with Elastica Masson’s staining. The CG was observed superior to the perineal membrane (arrowheads). The STPi was observed inferior to the perineal membrane, and the STPs and DTP were observed superior to the perineal membrane in the deep perineal pouch. (**B**) Immunostaining for striated muscle. The striated muscle fibers of the STPs covered the lateral side of the CG. (**C**) Immunostaining for smooth muscle. The DTP covered the superior side of the CG and consisted of smooth muscle fibers. Its smooth muscle fibers partially extended between the bilateral CGs and between their lobules (asterisk). *CG* Cowper’s gland, *DTP* deep transverse perineal muscle, *IPR* ischiopubic ramus, *LA* levator ani, *LM* longitudinal muscle, *STP* superficial transverse perineal muscle, *STPi* inferior bundle of the STP, *STPs* superior bundle of the STP.

Figure 5 includes coronal sections. The CG was observed superior to the perineal membrane (indicated by the arrowheads in Fig. 5A). Consistent with the macroscopic findings, the STP had superior and inferior muscle bundles. The STPi was observed inferior to the perineal membrane; the STPs and DTP were observed superior to the perineal membrane in the deep perineal pouch (Fig. 5A). The striated muscle fibers of the STPs covered the lateral side of the CG (Fig. 5A,B). In addition, the thin layer of the striated muscle wrapped not only the lateral side but also the superior and inferior sides of the CG. The DTP covered the superior side of the CG, and consisted of smooth muscle fibers (Fig. 5A,C). The DTP smooth muscle fibers partially extended between the bilateral CGs and between their lobules (indicated by the asterisk in Fig. 5C). Thus, these findings confirmed that the CG was surrounded laterally by the striated muscle of the STPs, and superomedially by the smooth muscle of the DTP.

### Three-dimensional reconstruction

**Figure 6:**
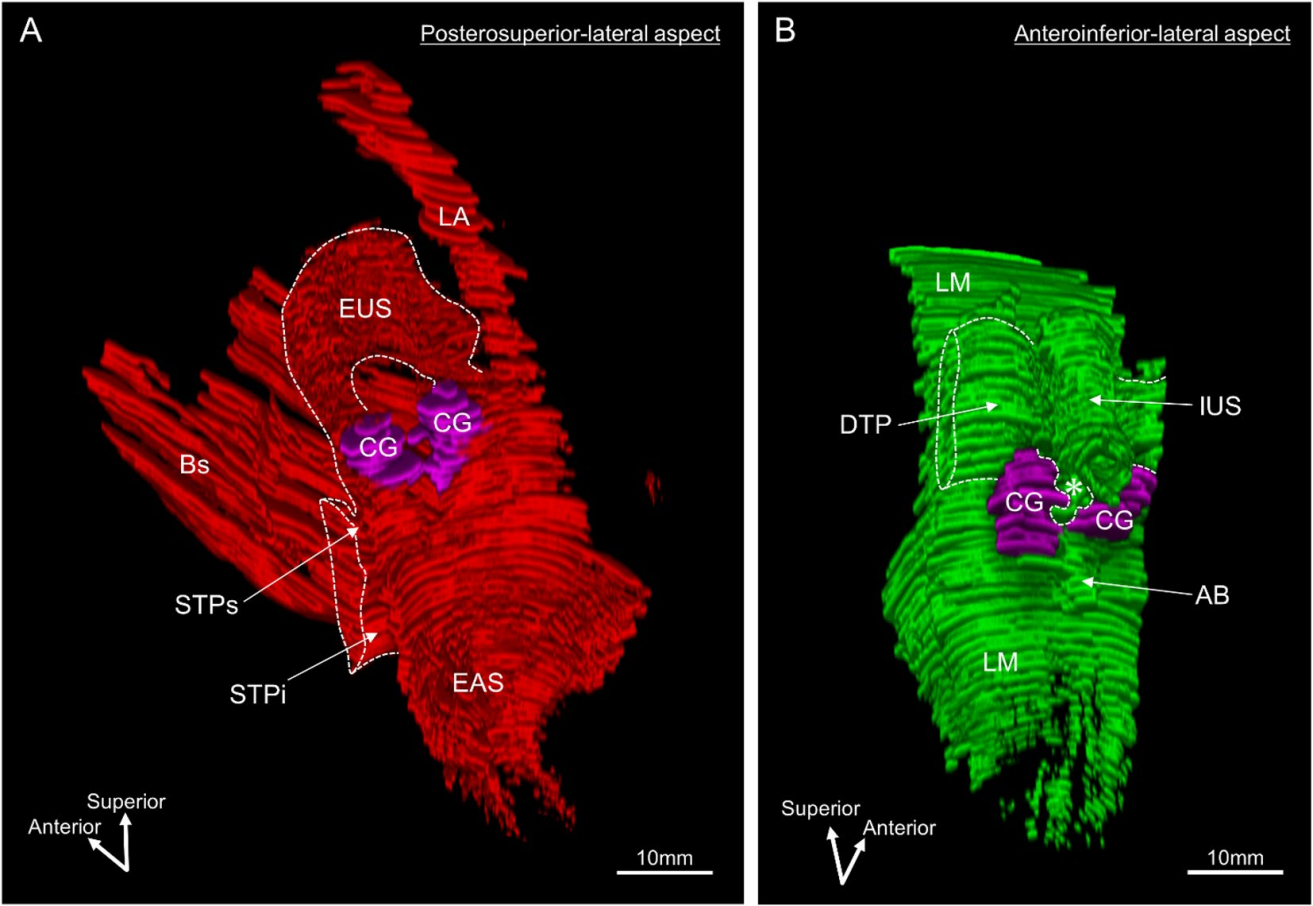
Three-dimensional reconstruction images showing the Cowper’s gland and surrounding muscles. (**A**) The CG (purple) and striated muscles (red) visualized from the posterosuperior-lateral aspect. The CG was laterally covered by the striated muscle of the STPs. The STPs was anterosuperiorly continuous with the EUS. The LA and EAS were continuous with the EUS. (**B**) CG (purple) and smooth muscles (green) visualized from the anteroinferior-lateral aspect. The DTP formed a plate-like wall posterosuperior to the CG. The area between the bilateral CGs was filled with smooth muscle continuous with the DTP (asterisk). The images were created using the SrfII software (ver. R.11.00.00.0-H, Ratoc, Tokyo, Japan, http://www.ratoc.com/eng/index.html). *AB* anterior bundle of the LM, *Bs* bulbospongiosus, *CG* Cowper’s gland, *DTP* deep transverse perineal muscle, *EAS* external anal sphincter, *EUS* external urethral sphincter (rhabdosphincter), *IUS* internal urethral sphincter, *LA* levator ani, *LM* longitudinal muscle, *STP* superficial transverse perineal muscle, *STPi* inferior bundle of the STP, *STPs* superior bundle of the STP.

The CG and surrounding striated and smooth muscles were observed by three-dimensional reconstruction (Fig. 6). The CG was laterally covered by the striated STPs muscle. The STPs was anterosuperiorly continuous with the EUS, which is a horseshoe-like shape (Fig. 6A). In addition, the LA and EAS were continuous with the EUS. The structure and location of the DTP were consistent with prior results (Fig. 6B). The area between the bilateral CG was filled with smooth muscle continuous with the DTP (indicated by the asterisk in Fig. 6B).

**Figure 7:**
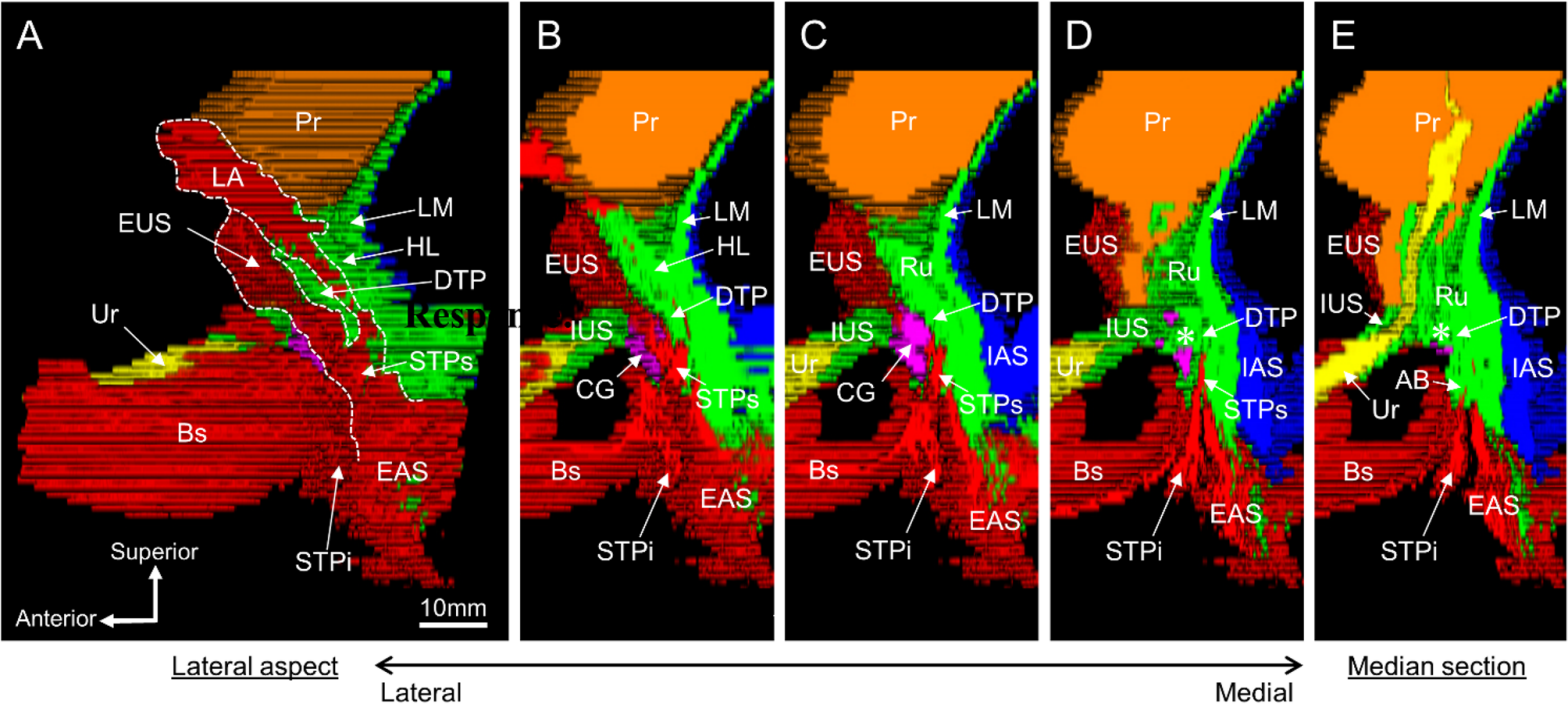
Three-dimensional reconstruction images showing the lateral aspect and sagittal sections. (**A**) The lateral aspect from left side. The LA, EAS, and STPs merged and extended anteriorly, forming the EUS on the lateral and anterior sides of the Ur. (**B**) A sagittal section lateral to the CG. The STPs was observed to cover the lateral side of the CG. The STPi was continuous with the Bs. (**C**) A sagittal section through the CG. The DTP was observed to cover the posterosuperior side of the CG. The region anteroinferior to the CG lacked striated muscle. (**D**) A sagittal section medial to the CG. The smooth muscle tissue continuous with the DTP entered medial to the CG (asterisk). (**E**) A median section. The EUS covered only anterior to the urethra. The region posterior to the urethra, between the urethra and rectum, was occupied by smooth muscles including the Ru, DTP, and AB. The images were created using the SrfII software (ver. R.11.00.00.0-H, Ratoc, Tokyo, Japan, http://www.ratoc.com/eng/index.html). *AB* anterior bundle of the LM, *Bs* bulbospongiosus, *CG* Cowper’s gland, *DTP* deep transverse perineal muscle, *EAS* external anal sphincter, *EUS* external urethral sphincter (rhabdosphincter), *HL* hiatal ligament, *IAS* internal anal sphincter, *IUS* internal urethral sphincter, *LA* levator ani, *LM* longitudinal muscle, *Pr* prostate, *STP* superficial transverse perineal muscle, *STPi* inferior bundle of the STP, *STPs* superior bundle of the STP, *Ur* urethra.

A three-dimensional image was observed from the lateral aspect, and the sagittal section was observed from lateral to medial (Fig. 7). In the lateral aspect, the LA, EAS, and STPs merged and extended anteriorly, forming the EUS on the lateral and anterior sides of the urethra (Fig. 7A). In the sagittal section lateral to the CG, the STPs was observed (Fig. 7B). In addition, the STPi was continuous with the Bs in this cross-section. In a sagittal section through the CG, the DTP was observed to cover the posterosuperior aspect of the CG (Fig. 7C). In this cross-section, the STPi and Bs were continuous, but the Bs was displaced inferiorly, and the region anteroinferior to the CG lacked striated muscle. In a sagittal section medial to the CG, the smooth muscle continuous with the DTP entered medial to the CG (indicated by the asterisk in Fig. 7D). The median section included a cross-section of the urethra, and the EUS covered only the anterior portion of the urethra (Fig. 7E). The region posterior to the urethra, between the urethra and rectum, was occupied by smooth muscle, including the rectourethralis, DTP, and the anterior bundle of the longitudinal muscle (Fig. 7E).

## Discussion

The CG in humans is surrounded laterally by striated muscle and posterosuperiorly by smooth muscles. The striated muscle was derived from the STP, LA, and EAS, and connected to the EUS, whereas the smooth muscle was part of the DTP and entered between the bilateral CGs and their lobules. This finding was consistently observed across all four samples that underwent histological examination, similar to the findings of macroscopic anatomy performed on three samples.

Several textbooks describe the CG in humans as being surrounded by the fibers of the EUS, which is a striated muscle^3–6^. A study on the EUS reported that it is difficult to analyze because its overall shape is unclear due to growth of the prostate and the invasion of the prostatic vascular plexuses, and that the textbook descriptions are inconsistent^7^. Although the term “sphincter” is reminiscent of a circular muscle, it has been reported that the EUS is horseshoe-shaped rather than perfectly circular^9,22,23^. Our group clarified that horseshoe-shaped EUS is not an independent muscle, but is formed from partial muscle bundles of the EAS, STP, and LA so it is continuous with the surrounding pelvic floor muscles^12^. On the other hand, a few textbooks and atlases often depict the CG as being buried in the DTP^4^. The DTP is a mysterious muscle; while there are reports that it does not exist^24,25^, some reports state that it is seen in 24.7%–100% of men and 16.7% of women^8,10,26–28^. The DTP is classically considered to be comprised striated muscle^4^; however, recent reports have indicated that it is comprised smooth muscle that traverses in the deep perineal pouch^11,16,29–32^.

The present study showed that the EUS surrounds the anterior and lateral sides of the urethra, and is formed from muscle bundles of the STPs, LA, and EAS extending into the deep perineal pouch. In addition, we clarified that this striated muscle bundle surrounds the lateral surface of the CG. Therefore, multiple striated muscles are likely involved in CG function. Although the CG is developmentally an appendage to the CSP, the Bs, which covers the CSP, is unlikely to be involved in CG function. We observed that the posterior part of the Bs did not completely cover the medial portion of the CSP, and was not in direct contact with the CG. It is interesting that the seemingly unrelated EAS, STP, and LA surround the CG while extending anteromedially. In contrast, the DTP was observed as a plate-like structure of smooth muscle located in the deep perineal pouch, in agreement with our recent report^11,16^. In the present study, we showed that the DTP surrounds the medial and posterosuperior parts of the CG. Therefore, we revealed additional details regarding the striated and smooth muscles that surround the CG in humans.

A recent physiological study reported that secretion from the CG involves somatic control of striated muscle, despite general acceptance that smooth muscle is involved in glandular secretion^2^. We can speculate the mechanism of secretion and emission from the CG in humans, based on muscle arrangement, composition, and continuity. The smooth muscle medial to the CG enters between the lobules of the CG and is likely responsible for secretion by contracting the lobules. This smooth muscle is characterized as part of a series of continuous smooth muscle structures in the pelvic floor^11,16^. It is noteworthy that smooth muscle, which is continuous with the longitudinal muscles of the rectum and the internal urethral sphincter, covers the medial side of the CG and extends between the lobules. Such smooth muscle continuity suggests that secretion from the CG facilitated by smooth muscle may occur in conjunction with movements of the rectum and urethra. Contrastingly, the striated muscle that covers the lateral side of the CG seems to compress the CG during contraction, causing emission. It is worth noting that this striated muscle is not a single independent muscle, but is comprised continuous muscle fibers from the EUS, STPs, EAS, and LA^12^. These pelvic floor and perineum muscles likely function in tandem. Due to muscle continuity, urethral closure induced by the EUS and emission from the CG seem to occur simultaneously. This action probably prevents secretions from refluxing into the proximal urethra on the bladder side, which is reasonable in terms of lubricating the lumen of the distal urethra. However, the striated muscle did not cover the entire circumference of the CG. When the CG is pulled backward by the STPs, LA, and EAS, the smooth muscle of the DTP creates a supportive wall behind the CG. This movement seems to compress the CG between the striated and smooth muscles, and could cause emission.

The present study has some limitations. First, it was purely an anatomical study; as such, we could not provide physiological experiments regarding secretion and emission from the CG. Second, the sample size was relatively small. Finally, the ages of the materials were imbalanced because the corpses of body donors used in this study were those of elderly adults with an average age of > 70 years.

## Conclusion

The CG in humans is surrounded by striated muscle on the lateral side and smooth muscle on the posterosuperior and medial sides. This striated muscle is part of a continuous structure stemming from the EUS, STPs, EAS, and LA muscle bundles, and the smooth muscle is the DTP, which is part of a series of continuous smooth muscle structures in the pelvic floor. These findings suggest that secretion and emission from the CG in humans is carried out through the cooperation of striated and smooth muscles.

## Data Availability

The data that support the findings of this study are available from the corresponding author upon reasonable request.
